# The opioid system in depression

**DOI:** 10.1016/j.neubiorev.2022.104800

**Published:** 2022-09

**Authors:** Luke A. Jelen, James M. Stone, Allan H. Young, Mitul A. Mehta

**Affiliations:** a Institute of Psychiatry, Psychology and Neuroscience, King’s College London, London, United Kingdom; b South London and Maudsley NHS Foundation Trust, London, United Kingdom; c Department of Neuroscience and Imaging, University of Sussex, United Kingdom

**Keywords:** Depression, Opioid system, Mu opioid receptor (MOR), Delta opioid receptor (DOR), Kappa opioid receptor (KOR), Nociceptin opioid receptor (NOP)

## Abstract

Opioid receptors are widely distributed throughout the brain and play an essential role in modulating aspects of human mood, reward, and well-being. Accumulating evidence indicates the endogenous opioid system is dysregulated in depression and that pharmacological modulators of mu, delta, and kappa opioid receptors hold potential for the treatment of depression. Here we review animal and clinical data, highlighting evidence to support: dysregulation of the opioid system in depression, evidence for opioidergic modulation of behavioural processes and brain regions associated with depression, and evidence for opioidergic modulation in antidepressant responses. We evaluate clinical trials that have examined the safety and efficacy of opioidergic agents in depression and consider how the opioid system may be involved in the effects of other treatments, including ketamine, that are currently understood to exert antidepressant effects through non-opioidergic actions. Finally, we explore key neurochemical and molecular mechanisms underlying the potential therapeutic effects of opioid system engagement, that together provides a rationale for further investigation into this relevant target in the treatment of depression.

## Highlights

• Opioid receptor subtypes, regulate mood-related processes including reward, motivation, and social behaviours.
• The opioid system is implicated in emotional processing and this system is dysregulated in depression.
• MOR/KOR agonist-antagonist combinations show early indication of antidepressant efficacy, while minimising the risk of abuse liability.
• DOR selective agonists and KOR selective antagonists, may be viable treatment options for MDD, without the risk of addiction.
• Further work is needed to understand the role of the opioid system in modulating the effects of existing antidepressants, such as ketamine.

## 1 Introduction

The major classes of antidepressant drugs include tricyclic and related antidepressants, monoamine oxidase inhibitors (MAOIs), serotonin-noradrenaline reuptake inhibitors (SNRIs) and selective serotonin re-uptake inhibitors (SSRIs). The newer classes of antidepressants were developed to target hypothesised deficits in monoaminergic neurotransmission, based on the fact that tricyclic antidepressants and MAOIs affect this system (Hirschfeld, 2000). There are important limitations to these treatment strategies, including a delay in the onset of action to produce remission of symptoms (from weeks to months), and with up to 30–40% of individuals with major depressive disorder (MDD) failing to demonstrate an adequate response, even following multiple medication exposures, leaving a significant proportion of individuals with persistent, treatment-resistant depression (TRD) (Sforzini et al., 2021, Al-Harbi, 2012). Crucially, treatment-resistance in MDD is associated with worse prognosis and increased levels of disability, morbidity, and mortality (Olchanski et al., 2013). There is therefore an urgent need for novel antidepressant treatment strategies.

For centuries, opioids have been used to treat a variety of physical health conditions, and historically opioids were also utilised in the treatment of a range of psychiatric disorders (Weber and Emrich, 1988). The so-called “opium cure” was widely used during the latter half of the 19th, up until the mid-20th century, and was noted as a rapidly effective treatment for refractory melancholic depression (Weber and Emrich, 1988, Tenore, 2008). Unfortunately, with the unregulated availability of opioid agents, the emergence of non-medical abuse of diverted supplies resulted in widespread dependence and addiction (White, 2014). With an increase in restrictive legislation surrounding their prescription, alongside the advent and availability of non-addictive antidepressant treatments in the 1950s, the use of opioids in the treatment of depression became obsolete. However, there is now a growing body of evidence that indicates the endogenous opioid system is directly involved in the regulation of mood and is dysregulated in depression (Berrocoso et al., 2009, Pecina et al., 2019), and this has led to a resurgence of interest in targeting the opioid system to alleviate depressive disorders (Browne et al., 2020).

In this review, we summarise the current literature implicating the opioid system in depression. First, we focus on preclinical evidence, examining individual opioid receptor systems as potential treatment targets for depression. Next, we consider experimental medicine studies that have highlighted dysregulated opioid signalling in the pathophysiology of depression before evaluating clinical studies that have emphasised the feasibility of targeting and modulating the opioid system in the treatment of MDD. In addition, we discuss potential opioidergic effects of compounds that are currently understood to exert antidepressant effects through non-opioidergic actions. Finally, we highlight some of the key neurochemical and molecular mechanisms that may underlie the potential therapeutic effects of opioid system engagement.

## 2 Endogenous opioid system

The endogenous opioid system is comprised of distinct opioid neuropeptides including β-endorphin, dynorphins, enkephalins and nociceptin/orphanin FQ, and four associated G-protein-coupled receptors (Benarroch, 2012). These include three classical opioid receptors: μ-opioid receptor [MOR], κ-opioid receptor [KOR] and δ-opioid receptor [DOR], and the non-opioid, nociceptin/orphanin FQ [NOP] receptor, encoded by the *Oprm1*, *Oprd1*, *Oprk1* and *Oprl1* genes respectively (Benarroch, 2012, Waldhoer et al., 2004). β-endorphin is the primary endogenous ligand for the MOR, however it also has agonist activity at the DOR and KOR. Dynorphins are endogenous peptides that primarily act on KORs, although have some affinity for the MOR and DOR. Met- and Leu-enkephalins are the endogenous ligands for the DOR but also show partial agonist activity at the MOR. Finally, nociceptin/orphanin FQ (N/OFQ) is the endogenous ligand for the NOP receptor (Waldhoer et al., 2004). The opioid system in humans plays a central role in pain processing and regulates other diverse aspects of physiology including respiratory, gastrointestinal, endocrine, and immune functions (Bodnar, 2020). Importantly, this system also regulates responses to stress and modulates aspects of human mood, reward, and well-being (Bodnar, 2020, Le Merrer et al., 2009). Endogenous opioid peptides and associated receptors are expressed in high concentrations in limbic and cortical brain regions implicated in the regulation of behavioural functions, including emotion, mood, motivation, and reward processing (Lutz and Kieffer, 2013, Peckys and Landwehrmeyer, 1999, Peng et al., 2012), that are characteristically altered in MDD. Increasing lines of evidence indicate the opioid system is directly involved in modulating these functions (Nummenmaa and Tuominen, 2018, Le Merrer et al., 2009) and therefore may represent a valuable target to explore in MDD research and drug development.

## 3 Preclinical evidence indicates role for opioid system in depression

Here, we summarise animal research data, including evidence from studies using genetic (receptor knockout) and pharmacological approaches, that have revealed opioid associations with processes and behaviours associated with depression and demonstrated effects of opioidergic drugs in rodent models of depression. It is important to acknowledge that these are only ‘models’; it is difficult to be confident about the human state that is being emulated by an abnormal behaviour in a particular model, and the translational validity of many of these models have been questioned (Stanford, 2020). As such, while animal work has shown promise in targeting the opioid system in depression, the inherent limitations should be kept in mind to avoid overinterpretation.

### 3.1 MOR

Opioid receptor knockout mice models have helped further our understanding of reward processing and mood state modifications, with relevance for mood disorders and addiction (Lutz and Kieffer, 2013). Studies of MOR knockout (Oprm1^-/-^) mice indicate an important role for MORs in mediating the rewarding and reinforcing properties of opioid and non-opioid drugs (Roberts et al., 2000, Ghozland et al., 2002, Nguyen et al., 2012a, Nguyen et al., 2012b, Becker et al., 2000), as well as the processing of natural rewards (Le Merrer et al., 2009). Through knockout mice models it has been proposed that MORs also play a key role in modulating social behaviours (Pellissier et al., 2018). In the context of natural reward processing, MOR knockout mice show both diminished food-anticipatory activity (Kas et al., 2004) and decreased motivation to eat (Papaleo et al., 2007). Regarding social behaviours, MOR knockout mice demonstrate reduced attachment behaviours (Moles et al., 2004), reduced interest in peers, with no preference for socially rewarding environments (Cinque et al., 2012), and reduced social interaction (Becker et al., 2014), suggesting MOR neurotransmission is fundamentally involved in the regulation of social hedonic capacity. As impairments in the domains of reward processing, motivation and social functioning are characteristically associated with MDD (Callaghan et al., 2018, Kupferberg et al., 2016), it follows that drugs that activate the MOR system may have antidepressant properties. This notion has been supported in the rodent learned helplessness (LH) paradigm (a model with behavioural phenotypes analogous to depressive symptoms (Maier and Seligman, 2016)), where administration of morphine, the prototypical MOR agonist, reversed the escape deficit induced by shock pre-treatment (Tejedor-Real et al., 1995, Besson et al., 1996). Administration of morphine to rodents also reduced immobility in the tail suspension test (TST) (Berrocoso et al., 2013) and forced swim test (FST) (Zomkowski et al., 2005). Furthermore, the effects of morphine in the FST were blocked by pre-treatment with the MOR antagonist naloxone (Zomkowski et al., 2005). While the FST (and TST) is a useful high-throughput predictive screen for antidepressant drugs, there is no evidence that immobility in the FST produces an animal ‘model’ of depression (Stanford, 2020, Planchez et al., 2019) and findings from these tests should not be overinterpreted. Other MOR agonists have also been shown to reverse escape deficits in the LH paradigm in rats (levorphanol, methadone and tramadol) (Rojas-Corrales et al., 2002) and reduce immobility in the TST in mice (codeine; levorphanol; methadone; tramadol) (Berrocoso et al., 2013). Finally, buprenorphine (a partial MOR agonist and KOR antagonist) reduces immobility in the FST in rats (Browne et al., 2015) and mice at 24 hrs after administration (Falcon et al., 2015). Importantly, in the study by Falcon et al. (2015), the KOR antagonist norbinaltorphimine (nor-BNI) also produced a similar effects in the FST at the 24-hr time point, but morphine was ineffective, suggesting the potential antidepressant-like effect of buprenorphine may be more closely related to its KOR antagonism rather than MOR agonist effects.

### 3.2 DOR

Investigation in DOR knockout mice (Oprd1^–/–^) has revealed that mice deficient for DORs display anxiogenic and pro-depressive behaviours (Filliol et al., 2000), highlighting a potential role for DORs in the regulation of mood states. DORs also appear to play a role in reward processing that is distinct from MORs (Le Merrer et al., 2009). Unlike MOR knockout mutants, DOR knockout mice show intact morphine reinforcement (Le Merrer et al., 2011), intact cannabinoid-induced reward (Ghozland et al., 2002), and increased ethanol self-administration (Roberts et al., 2001). In this study, as ethanol intake was sufficient to reverse the innate increased anxiety-like responses in this strain, it was hypothesised the increased ethanol consumption may be driven by the effect of DORs on anxiety-like behaviour (Roberts et al., 2001). Supporting a potential role for the endogenous DOR system in depression, administration of enkephalins reduced immobility in the FST (Kastin et al., 1978) and reversed escape deficits in the LH paradigm in rodents (Tejedor-Real et al., 1995). Furthermore, the enkephalinase inhibitor RB101 also reversed escape deficit’s in the LH (Tejedor-Real et al., 1998) and reduced immobility in the FST in rodents (Baamonde et al., 1992, Jutkiewicz et al., 2006b) that were blocked by naltrindole (a selective DOR antagonist). A number of DOR selective agonists were subsequently developed, demonstrating promising effects in rodents, reducing immobility in the FST (Broom et al., 2002b, Jutkiewicz et al., 2005), and attenuating the hyperemotional responses found in olfactory bulbectomized rats (Saitoh et al., 2008) (this rodent model exhibits behavioural abnormalities that may mimic the anxiety, aggression, and irritability seen in depression). However, some of these DOR agonists produce seizures/convulsions (Broom et al., 2002a, Jutkiewicz et al., 2006a), potentially limiting clinical applications. More recently, DOR selective agonists have been developed that demonstrate activity in the TST and FST rodent screening models for antidepressants (Naidu et al., 2007, Vergura et al., 2008, Saitoh et al., 2011), and do not produce convulsions, a property that may make these preferable candidate DOR-based antidepressant compounds.

### 3.3 KOR

The dynorphin-KOR system has been implicated as an endogenous anti-reward system (Lalanne et al., 2014). In contrast to the effects of MOR and DOR activation, KOR agonists have been shown to elicit pro-depressive- (Carlezon et al., 2006) anhedonia- (Todtenkopf et al., 2004), dysphoric- and aversive- like (Bruchas et al., 2007, Chefer et al., 2013, Anderson et al., 2014) behavioural effects in rodent models. While KOR knockout mice showed unchanged anxiety- and depressive-like behaviours (Filliol et al., 2000), mice with disruption of the gene coding for dynorphin showed no increase in immobility after exposure to a repeated FST (McLaughlin et al., 2003), suggesting chronic stress exposure may activate the KOR system to produce depressive-like effects. The findings that KOR activation resulted in behaviours suggestive of increased dysphoria, anhedonia and depression in rodents led to the evaluation of KOR antagonists in preclinical models of depression. KOR antagonists have consistently demonstrated positive effects in stress-naïve rodents in the FST antidepressant screening model (Mague et al., 2003, Beardsley et al., 2005, Grimwood et al., 2011, Rorick-Kehn et al., 2014).

KOR antagonists have been further evaluated for antidepressant-potential following chronic stress exposure paradigms that have greater translational application than experiments in stress-naïve animals (Jacobson et al., 2020a). In rodent studies utilising social defeat stress, pre-treatment with KOR antagonists prevented stress-related behaviours following exposure to social defeat (Grimwood et al., 2011, McLaughlin et al., 2006). Similarly, the KOR antagonist and partial MOR agonist buprenorphine, significantly reversed social interaction deficits produced by chronic social defeat stress following daily administration for 7 days (Browne et al., 2018). In another study, buprenorphine treatment normalised reductions in sucrose preference (decreased sucrose preference is widely considered a measure of anhedonia in rodents) following 3 weeks of unpredictable chronic mild stress (UCMS) (Falcon et al., 2016). A selective KOR antagonist (JNJ-67953964) has similarly been shown to reverse stress-induced deficits produced by UCMS on the sucrose preference test (Jacobson et al., 2020c). One potential benefit of selective KOR antagonists is that they may lack abuse liability as demonstrated by rodent studies showing they do not produce acutely rewarding or aversive effects in the conditioned place preference paradigm (Grimwood et al., 2011, Valenza et al., 2017). While buprenorphine has been shown to increase place preference in mice, this effect was blocked with naltrexone suggesting this is related to buprenorphine’s partial MOR agonist properties (Almatroudi et al., 2015).

Despite a narrative in the field that KOR agonists invariably produce pro-depressive behaviours in animal models, some studies report opposing results following administration of the KOR agonist salvinorin A. Indeed, salvinorin A administration resulted in reduced immobility in the FST and TST in rodents and an increased percentage of entries and time spent in open arms in the elevated plus maze (a test for putative anxiolytic compounds) in rodents (Braida et al., 2009). Furthermore, salvinorin A reversed suppressed sucrose preference following UCMS (a measure of anhedonia) (Harden et al., 2012). It has been argued that salvinorin A is an atypical KOR agonist, with a complex pharmacological profile, influencing dopaminergic, glutamatergic and cannabinoid systems, and should be examined as a potential treatment for depression (Taylor and Manzella, 2016).

### 3.4 NOP receptor

The NOP receptor is expressed in widespread areas of the central nervous system in rodents (Neal et al., 1999), non-human primates (Kimura et al., 2011), and humans (Lohith et al., 2012), in various regions including the cortex, hippocampus, amygdala, thalamus and dorsal raphe nucleus which are all associated with mood disorders. N/OFQ modulates a range of processes including pain, learning and memory functions and pathways associated with stress, depression, and anxiety (Gavioli and Calo, 2013). Furthermore, increasing evidence has revealed interactions of N/OFQ, the NOP receptor and the classical opioid receptors (MOR, DOR and KOR) which may modulate their function (Donica et al., 2013). Preclinical work demonstrated that NOP receptor knockout mice show reduced immobility time in the FST, and administration of selective NOP receptor antagonists also reduces immobility time in this assay (Redrobe et al., 2002, Gavioli et al., 2003). Additional work found chronic treatment with the selective NOP receptor antagonist (UFP-101) in rodents exposed to UCMS reinstated sucrose consumption in stressed animals, restored stress-induced monoamine level alterations and abolished increases in serum corticosterone (Vitale et al., 2009). These behavioural and biochemical effects mimicked those of the reference antidepressant drug imipramine. Together, preclinical evidence has supported the hypothesis that blockade of NOP receptor signalling could be another innovative strategy in the development of novel antidepressants (Gavioli and Calo, 2013).

## 4 Experimental medicine studies support role for opioid system in depression

While preclinical evidence has highlighted opioid associations with key processes associated with depression and positive effects of opioidergic drugs in rodent models of depression, there is always the need for caution in generalising findings from rodent models to humans (Harro, 2019, Stanford, 2020). Of particular relevance to the study of the opioid system, although opioid receptors follow a similar distribution pattern, there is relatively more KOR binding and relatively less DOR in humans compared with rodents (Pfeiffer et al., 1982). Furthermore, there are potential species differences in terms of KOR (Schattauer et al., 2012) and MOR (DeWire et al., 2013, Schmid et al., 2017) ligand effects on downstream signalling pathways. Therefore, experiments carried out in humans are necessary to help translate preclinical findings and better understand how the opioid system may regulate emotions, mood, and social behaviours relevant to the potential role of opioids in depression and its treatment.

### 4.1 MOR

#### 4.1.1 Emotional processing

The distribution of MORs can be quantified in vivo with positron emission tomography (PET), using the MOR-selective radiotracer [^11^C]‐carfentanil. PET studies suggest that the distribution of MORs in the human brain shares a significant overlap with brain regions (Kuhar et al., 1973) and circuits (Nummenmaa and Tuominen, 2018) implicated in emotional processing and regulation. PET studies involving experimental paradigms have also been utilised to explore endogenous opioid neurotransmission during emotional processing, with increases and decreases in [^11^C]-carfentanil binding potential indexing decreases and increases in MOR-mediated neurotransmission respectively. In one such PET study in fourteen healthy female volunteers, the self-induction of a sustained sadness state was associated with significant reductions in MOR-mediated neurotransmission in the rostral anterior cingulate (rACC) (Zubieta et al., 2003), a region implicated in the regulation of mood, cognitive control and emotional processing (Stevens et al., 2011, Bush et al., 2000). Furthermore, reductions in MOR neurotransmission in the rACC significantly correlated with increases in negative affect ratings during the sadness state (Zubieta et al., 2003). A subsequent PET study also found reduced MOR neurotransmission, localised in the rACC, during a sustained sadness state in healthy female controls (n = 14) that correlated with negative affect scores during the sadness state (Kennedy et al., 2006). Conversely, in matched patients diagnosed with MDD; the same sadness condition was associated with an increase in MOR-mediated neurotransmission in the left inferior temporal cortex which correlated with negative affect ratings during the sadness state. MDD patients that did not respond to SSRI treatment demonstrated increases in rACC MOR neurotransmission during sadness induction, while treatment responders demonstrated reduced MOR neurotransmission in this region, similar to healthy controls (Kennedy et al., 2006). Direct comparisons between MDD patients and controls in the neutral state showed significantly lower MOR binding potential in the thalamus (Kennedy et al., 2006), supporting the hypothesis of an overactivation (or alternatively, a down-regulation) of these receptors in MDD. However, as this study used a radiotracer displacement paradigm, the interpretation of such baseline differences is challenging. Nevertheless, in support of preliminary findings by Kennedy et al., a recent, large-scale retrospective cross-sectional study (pooled data, n = 135) demonstrated that subclinical depressive and anxious symptoms were consistently associated with lower MOR availability across cortical and subcortical regions including the amygdala, insula, hippocampus, ventral striatum, orbitofrontal and cingulate cortices (Nummenmaa et al., 2020). Taken together, these studies highlight a potential role for dysregulated MOR availability in altered mood and in the pathophysiology of depression.

#### 4.1.2 Social behaviours

Additional PET work has explored the involvement of the MOR system in regulating social rejection and acceptance in healthy and depressed subjects (Hsu et al., 2013, Hsu et al., 2015). Importantly, rejection-related stressors are predictive of MDD (Kendler et al., 2003, Keller et al., 2007), dysfunctional emotional processing in response to rejection may contribute to depression (Kupferberg et al., 2016) and the reduced pleasure individuals with MDD experience from social interactions (social anhedonia) can also perpetuate depression (Kupferberg et al., 2016). As social rejection and physical pain share common neural pathways (Kross et al., 2011), the theory of “social pain” suggests that responses to social rejection may be regulated by the MOR system in a similar manner to physical pain (Eisenberger, 2012, Lutz et al., 2020). Hsu and colleagues first tested this hypothesis in healthy volunteers (n = 18) by examining MOR availability with PET during a social feedback task with social rejection and acceptance conditions (Hsu et al., 2013). Social rejection led to significant reductions in [^11^C]-carfentanil binding, interpreted as “MOR activation”, in regions involved in emotional regulation including the ventral striatum, amygdala and thalamus (Hsu et al., 2013); a pattern similar to that seen during physical pain (Zubieta et al., 2001). Furthermore, greater trait resiliency was associated with MOR activation in the amygdala, periaqueductal grey and subgenual anterior cingulate cortex (sgACC) during rejection, suggesting a protective or adaptive role for MOR activation in these areas (Hsu et al., 2013). In a follow up PET study using the same task, during rejection, MDD patients (n = 17) showed reduced MOR activation in the nucleus accumbens, amygdala and midline thalamus, and slower emotional recovery compared to healthy controls (n = 18) (Hsu et al., 2015). During acceptance, only healthy controls showed increases in the desire for social interaction, and this was positively correlated with MOR activation in the nucleus accumbens, a primary site mediating reward behaviour. In patients with MDD, increased [^11^C]carfentanil binding in the nucleus accumbens (suggesting reduced MOR neurotransmission) was observed during the acceptance condition (Hsu et al., 2015). These studies suggest that abnormal MOR function in MDD may both contribute to dysfunctional emotional processing of negative social interactions but also reduce the rewarding aspects of positive social interactions.

Several studies have explored the impact of MOR genetic variations on social behaviours and depressive symptoms with a focus on the A118G single nucleotide polymorphism (SNP) in the MOR (OPRM1) gene, where G allele status has been associated with loss of receptor function (Zhang et al., 2005, Kroslak et al., 2007). Findings suggest minor G allele carriers show greater neural responses to social rejection in regions implicated in the processing of social pain (dorsal anterior cingulate and anterior insula) (Way et al., 2009), are more likely to manifest withdrawn behaviours in response to social rejection (Bertoletti et al., 2012), and show reduced MOR binding in brain regions implicated in affective regulation (subgenual, rostral and dorsal anterior cingulate cortex, nucleus accumbens and thalamus) (Pecina et al., 2015b). Furthermore, in a large longitudinal birth cohort study (n = 420), G allele carriers were more severely depressed and twice as likely to meet criteria for MDD after experiencing a rejection major life event relative to A/A homozygotes who experienced similar stressors (Slavich et al., 2014). Although these studies suggest G allele carriers may be particularly sensitive to social rejections and vulnerable to depressive symptoms, potentially mediated by reduced MOR expression/activity, single SNP analyses yield high false-positive rates (Udden et al., 2019) and do not address the polygenic architecture of psychiatric disorders, and should therefore be interpreted cautiously (Dima and Breen, 2015).

#### 4.1.3 Placebo effects

There is accumulating evidence demonstrating that the endogenous opioid system, when activated by positive individual expectations and maintained by subsequent conditioning and reward learning may induce ‘internally mediated’ changes in physiological effects (the placebo effect) resulting in the experience of analgesia and improvements in emotional state (Pecina and Zubieta, 2015). While earlier pharmacological and neuroimaging work has focussed on highlighting the role of the opioid system in placebo analgesia (Levine et al., 1978, Petrovic et al., 2002, Zubieta et al., 2005), more recently the opioid system has also been implicated in the formation of antidepressant placebo effects (Pecina et al., 2015a, Pecina et al., 2018, Pecina et al., 2021).

In a 2-week single-blinded, crossover, RCT of two identical oral placebos (described as having either active or inactive fast-acting antidepressant-like effects) followed by 10-weeks of open-label antidepressant treatment, 35 medication-free MDD patients were studied with [^11^C]‐carfentanil PET after each 1-week inactive and active oral placebo treatment (Pecina et al., 2015a). During the PET scan, but only after the active placebo condition, participants were administered IV saline, with instructions the compound may be associated with the activation of brain systems involved in mood improvement. Higher baseline MOR binding in the nucleus accumbens was associated with a better response during the open label antidepressant treatment and reductions in depressive symptoms after 1 week of active placebo treatment (compared with inactive) were associated with placebo-induced MOR neurotransmission in a network of regions implicated in emotion, stress regulation, and the pathophysiology of MDD (sgACC, nucleus accumbens, thalamus, and amygdala). Furthermore, placebo-induced endogenous opioid release in these regions was associated with better treatment response, predicting 43% of the variance in symptom improvement at the end of the antidepressant trial (Pecina et al., 2015a). Subsequent work utilised an acute antidepressant placebo experiment involving IV administration of a “fast-acting antidepressant” (expectancy manipulation) and a trial-by-trial sham fMRI “neurofeedback” manipulation, purportedly revealing mood improvement (conditioned reinforcement) in 20 patients with MDD (Pecina et al., 2018). Infusion cues induced higher expectancies of mood improvement, and both the infusion cue and the sham neurofeedback induced a reported mood improvement. The expectancy and reinforcement manipulation of antidepressant placebo effects resulted in increased BOLD responses in the lateral PFC (vlPFC and dlPFC) and placebo-induced increases in plasma levels of β-endorphin were associated with higher expectancy and mood ratings (Pecina et al., 2018). Recently, Pecina and colleagues utilised a modified version of this fMRI paradigm in 20 medication-free MDD patients, in a randomised double-blind crossover study comparing a single oral dose of naltrexone 50 mg to matching placebo immediately before the task (Pecina et al., 2021). At the behavioural level, naltrexone partially abolished the interaction of the expectancy and reinforcement condition on expectancy and mood ratings (i.e., the antidepressant placebo effects). At the neural level, naltrexone blocked reinforcement-induced responses in the right central orbitofrontal cortex (OFC) (Pecina et al., 2021), a brain region abundant in MORs (Mansour et al., 1995) that appears to have a crucial role for signalling outcome expectancies to guide behaviour (Schoenbaum et al., 2009). Together, these findings suggest a role of mu-opioid central OFC modulation in antidepressant placebo effects based on reinforcement and reward associations (Pecina et al., 2021). Speculative implications are that MOR antagonists could be applied to reduce placebo effects in antidepressant clinical trials or that modulation of the mu-opioid system could be a target to enhance antidepressant expectancy effects and clinical response.

### 4.2 DOR and KOR

Despite evidence linking DOR and KOR signalling to depression-like symptoms from preclinical work, compared to the MOR, the DOR and KOR systems remain relatively understudied in humans with mood disorders.

A selective DOR antagonist [^11^C]-methyl-naltrindole is available and using this PET radiotracer the distribution of DORs has been examined in healthy humans (Madar et al., 1996). [^11^C]-methyl-naltrindole binding was found to be highest in regions of the neocortex (insular, parietal, frontal, cingulate and occipital), caudate nucleus and putamen, with intermediate binding in the amygdala (Madar et al., 1996). Although the localisation of DORs to some of these regions may be consistent with a role in regulating mood, this is speculative, and studies are yet to apply [^11^C]-methyl-naltrindole (or other DOR selective antagonists) to examine DOR distribution/density in patients with depression.

Several KOR radioligands are available for use in humans, although most of the PET work quantifying KOR has been in healthy controls (Naganawa et al., 2016, Naganawa et al., 2014, Naganawa et al., 2015). One pilot PET study utilising the selective KOR agonist radiotracer [^11^C]-GR103545 radiotracer that compared volume of distribution (V_T_) of the tracer that was estimated regionally as a measure of KOR availability in 13 healthy volunteers and 10 participants with current MDD (Miller et al., 2018). In this study there were no differences in [^11^C]-GR103545 V_T_ in four a priori regions of interest (nucleus accumbens, amygdala, hippocampus, and raphe nuclei) nor were there any significant relationships between [^11^C]-GR103545 V_T_ and measures of life stress or depression severity. However, limitations of this study include the small sample size, low specific binding, slow kinetics of [^11^C]-GR103545 and poor test-retest reliability of [^11^C]-GR103545 V_T_ (Miller et al., 2018). More recently, it has been found that a novel selective KOR agonist [^11^C]-EKAP shows faster tissue kinetics than [^11^C]-GR103545, requires a shorter minimum scan time and provides excellent test-retest reproducibility of regional *V*_T_, making it a better tracer for imaging and quantification of KOR in humans (Naganawa et al., 2020). PET work using the [^11^C]-EKAP radiotracer has investigated the effect of social status on the KOR system in healthy participants and found that lower social status was associated with increased KOR levels in the reward/aversion areas of the amygdala, anterior cingulate cortex (ACC), caudate, frontal cortex, hippocampus, pallidum, putamen, and ventral striatum (Matuskey et al., 2019). With lower social status viewed as a socioeconomic stressor, the authors suggest the KOR system may act as a mediator for the negative effects of social stressors in humans that could have implications in depression and other psychiatric disorders (Matuskey et al., 2019). Further work with selective KOR radiotracers in larger clinical population samples is needed to advance our understanding of the KOR system in depression.

### 4.3 NOP receptor

Additional human data supports a role for the N/OFQ – NOP receptor system in depression with studies reporting increased N/OFQ plasma levels in patients with post-partum depression (Gu et al., 2003) and elevated levels in patients with bipolar depressive disorder that positively correlated with level of depressive symptoms (Wang et al., 2009). Selective radioligands for imaging brain NOP receptors with PET, including ^11^C-NOP-1A and ^18^F-MK-0911, have been developed (Lohith et al., 2012, Pike et al., 2011). ^11^C-NOP-1A PET has demonstrated regional distribution in the healthy human brain with cortical and subcortical structures such as the caudate and thalamus having higher uptake (Lohith et al., 2014), however studies are yet to apply ^11^C-NOP-1A to examine NOP receptor density in depressed patients. Nonetheless, the ^11^C-NOP-1A tracer has proven useful for the assessment between NOP receptor occupancy, dose, and plasma concentration of the NOP antagonist LY2940094, helping to determine an appropriate dosing strategy (40 mg/daily) to achieve sustainably high NOP receptor occupancy suitable to test clinical efficacy (Raddad et al., 2016).

Experimental medicine studies have shown that patients with depression are more likely to interpret emotional signals such as facial expressions as being negative (or less positive) compared with healthy individuals and it has been proposed that antidepressants may work by reversing negative biases in depressed patients before the alterations in mood (Harmer et al., 2011). In an 8-week proof-of concept study patients with MDD were randomised to receive LY2940094 at 40 mg versus placebo it was found that LY2940094 was associated with a greater accuracy of identifying positive faces as compared with placebo at Week 1 (Day 7) on the Facial Expression Recognition Task (FERT) (Post et al., 2016), consistent with effects observed with established antidepressant drugs (Harmer et al., 2004). Furthermore, the accuracy in recognising happy facial expressions correlated with eventual treatment response to the NOP receptor antagonist but not to placebo treatment, measured by the Hamilton Depression Rating Scale 7 weeks later (Dawson et al., 2021). Overall, these are the first human data providing evidence that blockade NOP receptor signalling may represent a promising treatment strategy for MDD.

## 5 Clinical studies of opioidergic agents in depression

A summary of the studies described in this section is provided in Table 1. Early clinical studies performed from the late 1970 s first explored the potential antidepressant effects of IV infusions of synthetic β-endorphin (a MOR ligand) in the treatment of depression (Kline et al., 1977, Angst et al., 1979, Gerner et al., 1980, Pickar et al., 1981). While these early reports were limited by small patient numbers, inconsistent dosing strategies and limited control conditions, the majority suggested rapid-antidepressant effects occurring within hours of administration (Kline et al., 1977, Angst et al., 1979, Gerner et al., 1980). Notably, in one double-blind placebo-controlled crossover study in 10 subjects with either MDD (7), bipolar depression (2) or schizoaffective disorder (1), a single IV infusion of β-endorphin in doses ranging from 1.5 to 11.5 mg led to a significant improvement in depressive symptoms compared with placebo 2–4 hrs after the infusion (Gerner et al., 1980). Other early studies also investigated potential antidepressant effects of synthetic opioid drugs including morphine, methadone, and codeine in MDD (Extein et al., 1981, Varga et al., 1982). While neither of these studies demonstrated convincing antidepressant effects for any of these agents, methodological shortcomings (including small sample sizes and inadequate control conditions), make it difficult to draw firm conclusions (Extein et al., 1981, Varga et al., 1982). Furthermore, morphine, methadone, and codeine all have significant potential for abuse, dependence and addiction (Kosten and George, 2002) which is a significant limitation in considering any antidepressant therapeutic potential.

**Table 1:** Summary of clinical studies of opioidergic agents in major depressive disorder.

STUDY	DRUG	DESIGN	PATIENT GROUP	(N)	DOSING REGIME	MAIN OUTCOME	ANTIDEPRESSANT EFFECTS
Kline et al. (1977)	β-Endorphin	Single-blind, placebo controlled	MDD (2) BD (1)	3	1.5 −10 mg IV β-Endorphin on two separate occasions	Clinician-rated improvement	-Rapid antidepressant response observed within minutes after first injection but not following repeat dosing.
Angst et al. (1979)	β-Endorphin	Open-label	MDD (2) BD (4)	6	10 mg IV β-Endorphin over 5 min	Self-rated visual analogue scales	-Change of depressive symptoms observed in all patients within the first 20–30 min. There was an initial increase of energy and mood and a decrease of anxiety, depression and restlessness. -Changes persisted in general for 2 h but were not maintained.
Gerner et al. (1980)	β-Endorphin	Double-blind, placebo-controlled crossover	MDD (7) BD (2) Schizoaffective- depressed (1)	10	1.5 – 11.5 mg IV β-Endorphin over 30–35 min	^Modified Bunney-Hamberg scale^	-Significant improvement in depressive symptoms (P = 0.05) after β-endorphin compared with placebo 2–4 hrs after infusion. -Proportion of depressed patients whose condition improved was significantly greater after β-endorphin than placebo (P < 0.025).
Pickar et al. (1981)	β-Endorphin	Double-blind, placebo-controlled crossover	MDD	4	4–10 mg IV β-Endorphin over 5 min	^-Brief Psychiatric Rating Scale (BPRS)^	-No significant difference in ratings between β-Endorphin and placebo.
Extein et al. (1981)	Morphine	Open-label	MDD (9), BD (1)	10	5 mg IV Morphine	-Self-rated mood	-Small nonsignificant antidepressant and antianxiety effects in both the depressed and control groups.
	Methadone	Double-blind, placebo-controlled crossover	MDD (2), BD (4)	6	5 mg IV Methadone	-Self-rated mood and BPRS	-3/6 reported themselves "better" two the "same," and one "worse" 30 min after methadone infusion, whereas all 6 reported themselves the "same" 30 min after saline infusion. -Methadone infusion produced little change according to BPRS.
Varga et al. (1982)	Codeine	Open-label	MDD	12	Oral codeine 90 mg/day to 180 mg/day for 3 weeks monotherapy (4) or added to tricyclic antidepressant (8)	-HAM-D -Zung depression self-rating scale	-4 depressed patients received codeine alone and none of them improved. -8 patients received codeine in combination with other TCAs and only one of them showed improvement.
Emrich et al. (1982)	Buprenorphine	Double-blind, placebo-controlled	MDD	10	Sublingual 0.2 mg buprenorphine for 5–8 days	HAM-D	-Significant reduction in HAM-D scores (P < 0.02) during buprenorphine treatment compared with placebo. -50% of the patients responded very strongly to buprenorphine, whereas the other 50% were non responders.
Mongan and Callaway (1990)	Buprenorphine	Some subjects open label, others double-blind.	MDD (7) BD (1)	8	Sublingual 0.15 – 0.3 mg buprenorphine	Profile of Mood Scale (POMS)	-6/8 demonstrated antidepressant response lasting six hours or longer.
Bodkin et al. (1995)	Buprenorphine	Open-label	MDD (TRD)	10	Buprenorphine 0.15 mg initiated intranasally or sublingually once daily titrated according to tolerance and clinical benefit, with a maximum daily dosage of 1.8 mg for 4 weeks	HAM-D	-6/7 subjects achieved marked clinical improvement by the end of the trial (3 dropped out). -Mean endpoint HAM-D score was 10.7 (SD = 9.3), representing a 60.7% reduction from baseline (p = 0.006).
Nyhuis et al. (2008)	Buprenorphine	Open-label	MDD (TRD)	6	Sublingual buprenorphine as monotherapy beginning with 0.4 mg/d increased up to final dosage of 0.8–2.0 mg once daily for 1 week	HAM-D	-HAMD score decreased within 1 week of buprenorphine treatment from an average of 22.8 (range, 17–28) to 6.0 (range, 2–10). -5/ 6 patients reached complete remission.
Karp et al. (2014)	Buprenorphine	Open-label	MDD (TRD)	15	Titrated dose of sublingual buprenorphine ranged from 0.2 to 1.6 mg/d for 8 weeks	MADRS	-MADRS at baseline was 27.0 (SD = 7.3) and at week 8 was 9.5 (SD = 9.5). -Response at the end of 8 weeks was observed for 8/13 participants (61.54%, 95% CI = 32.3–84.9).
Ehrich et al. (2015)	Buprenorphine + Samidorphan *(adjunct to antidepressant)*	Randomised double-blind placebo-controlled study	MDD (TRD)	32	-BUP/SAM 8: 1 dose-ratio (2 mg/0.25 mg for 3 days and 4 mg/0.5 mg for 4 days) (*n* = 14) -BUP/SAM 1: 1 dose-ratio (4 mg/4 mg for 3 days and 8 mg/8 mg for 4 days) (*n* = 14) -Placebo (*n* = 4)	HAM-D	-Antidepressant effects were greatest in 1:1 ratio group. -For the 1:1 treatment group, the difference in HAM-D from placebo was significant (p = 0.032).
Yovell et al. (2016)	Buprenorphine *(monotherapy or adjunct to antidepressant)*	Randomised double-blind placebo-controlled trial	Severely suicidal patients (average baseline score on the Beck Suicide Ideation scale (BSI), 19.7).	88	-Sublingual buprenorphine (initial dosage, 0.1 mg once or twice daily; mean final dosage=0.44 mg/day; n = 57) for 4 weeks -Placebo (n = 31)	-BSI -BDI	-Buprenorphine group had a greater reduction in BSSI than placebo at week 2 (mean difference=24.3, 95% CI=28.5, 20.2; p = 0.04) and at the end of week 4 (mean difference=27.1, 95% CI=212.0, 22.3; p = 0.004). - Buprenorphine group had lower BDI scores than the placebo group, although difference did not reach significance (mean difference=26.1, 95% CI=213.2, 1.0; p = 0.09).
Fava et al. (2016)	Buprenorphine + Samidorphan *(adjunct to antidepressant)*	Randomised double-blind placebo-controlled trial	MDD (TRD)	142	-BUP/SAM 2 mg/2 mg; (n = 24) -BUP/SAM 8 mg/8 mg (n = 19) -Placebo (n = 98) -4 weeks	-HAM-D -MADRS -CGI	-Significantly greater improvements in the 2/2 dosage group across the outcome measures compared with placebo (HAM-D: −2.8, 95% CI=−5.1, −0.6; MADRS: −4.9, 95% CI=−8.2, −1.6; CGI-S: −0.5, 95% CI=−0.9, −0.1).
Zajecka et al. (2019)	Buprenorphine + Samidorphan *(adjunct to antidepressant)*	Phase 3 multicentre randomised, placebo-controlled study conducted at 58 study sites	MDD (TRD)	295	-BUP/SAM 2 mg/2 mg (n = 147) -Placebo (n = 148) -6 weeks	MADRS	-Least-squares mean change in MADRS at end of treatment was − 4.8 (SE 0.67) in the BUP/SAM 2 mg/2 mg group and − 4.6 (SE 0.66) in the placebo group (mean difference −0.3 [SE 0.95], P = 0.782). -There were no differences in MADRS-based response or remission rates.
Fava et al. (2020b)	Buprenorphine + Samidorphan *(adjunct to antidepressant)*	Two phase 3, randomized, double-blind, placebo-controlled studies *FORWARD-4 (**FW4**)* *FORWARD-5 (**FW5**)*	MDD (TRD)	***FW4:*** 168 ***FW5:*** 187	-BUP/SAM (0.5 mg/0.5 mg) (*n* = 56) -BUP/SAM (2 mg/2 mg) (*n* = 56) -Placebo (n = 56) -BUP/SAM (1 mg/1 mg) *(n* = 62) -BUP/SAM (2 mg/2 mg) (*n* = 63) -Placebo (n = 62) -6 weeks	MADRS	-**FW5**: Adjunctive BUP/SAM 2 mg/2 mg was superior to placebo (average difference change from baseline to week 6 in MADRS versus placebo: −1.9, P = 0.026). -**FW4**: Did not achieve the primary endpoint (change from baseline in MADRS-10 at week 5 versus placebo: –1.8, P = 0.109). -In pooled analysis, change from baseline on the MADRS in the BUP/SAM 2 mg/2 mg group was greater compared with placebo at all timepoints from week 3 and later, including week 6 (–1.8; P = 0.010; 95% CI: –3.2 to –0.4).
Fava et al. (2020a)	CERC-501 (JNJ-67953964) (a kappa selective opioid receptor antagonist)	Sequential Parallel Comparison Design study	MDD (TRD)	8	a) 10 mg/d of CERC-501 for 6 days b) 20 mg/d of CERC-501 for 6 days c) placebo for 3 days followed by 10 mg/d of CERC- 501 for 3 days d) placebo for 3 days followed by 20 mg/d of CERC-501 for 3 days e) placebo for 6 days	HAMD-6 MADRS	-Study terminated early due to slow enrolment and sample size limits the ability to draw conclusions. -Weighted mean difference of changes (drug vs placebo) in the HAMD-6 (1.28) and (MADRS) (2.33) were all numerically but not statistically greater for CERC-501 than for placebo.
Richards et al. (2016)	AZD2327 (a selective DOR agonist)	Double-blind, parallel group design, placebo-controlled pilot study	Anxious MDD	22	-AZD2327 3 mg BD (n = 13) -Placebo (n = 9) -4 weeks	HAM-D HAM-A	-Seven (54%) patients responded to active drug and three (33%) responded to placebo. -No significant main drug effect was found on either the HAM-D (p = 0.39) or the HAM-A (p = 0.15).
Post et al. (2016)	LY2940094 (a selective NOP receptor antagonist)	Double-blind, parallel group design, placebo-controlled proof-of-concept study	MDD		- LY2940094 (n = 68) -Placebo (n = 61) -8 weeks	HAM-D	-At Week 8, least-squares mean changes from baseline in HAMD total scores were − 11.4 and − 9.8 for patients in the LY2940094 and placebo treatment groups, respectively. The LS mean difference from placebo was − 1.5 (95% CI − 4.7, 1.7). -The probability that LY2940094 was better than placebo was 82.9%, which was close to, but did not meet, the pre-defined proof-of-concept criterion (88% probability).

Several subsequent studies have evaluated the antidepressant effects of the MOR partial agonist, KOR antagonist, buprenorphine in MDD (Emrich et al., 1982, Mongan and Callaway, 1990, Bodkin et al., 1995, Nyhuis et al., 2008, Karp et al., 2014). While all of these studies had a small sample size (n = 6–15), and only one included a placebo control condition (Emrich et al., 1982), antidepressant effects were consistently reported following low doses of sublingual or intranasal buprenorphine ranging from 0.15 mg to 2.0 mg/day, including in patients with TRD. Further work has examined whether buprenorphine may have specific anti-suicidal effects (Yovell et al., 2016). In a randomised double-blind placebo-controlled trial in patients with severe suicidal ideation (including individuals with MDD, borderline personality disorder, adjustment disorder, eating disorders, post-traumatic stress disorder and bipolar disorder), sublingual buprenorphine at a mean dose of 0.44 mg/day led to a significantly greater reduction in suicidal symptoms to placebo at both 2-weeks and the 4-week primary endpoint (Yovell et al., 2016). Concurrent use of antidepressants and a diagnosis of borderline personality did not affect the anti-suicidal response to buprenorphine. Even though buprenorphine is only a partial MOR agonist with a much lower potential for dependence and misuse than pure agonists like morphine, it still has abuse potential, and some people still misuse the drug (Chilcoat et al., 2019).

Although increasing clinical evidence has demonstrated beneficial effects of opioid agents in depression, the widespread use of treatments acting as full or partial MOR agonists in MDD has been limited by major concerns regarding the risk of abuse and dependency. To address this, the combination of buprenorphine and a potent MOR antagonist, samidorphan, (BUP/SAM) has been investigated to determine whether antidepressant effects may still be achieved through opioid modulation while avoiding the potential for addiction. In the first RCT by Ehrich et al. (2015), comparing two different dose ratios of BUP/SAM (8:1 and 1:1) versus placebo in TRD, after one week of treatment the greatest antidepressant effect was seen in the 1:1 ratio group that significantly outperformed placebo. The robust antidepressant effect of the 1:1 ratio reported in this study suggests that greater MOR agonist activity may not be linked to greater antidepressant effects. In another larger RCT, the 1:1 ratio combination of BUP/SAM (2 mg/2 mg a day) over a 4-week treatment period, again led to significantly greater antidepressant effects than placebo (Fava et al., 2016). In subsequent phase 3 TRD studies, the BUP/SAM combination has not consistently outperformed placebo. In the FORWARD-3 trial, although improvements in MADRS scores in the BUP/SAM (2 mg/2 mg a day) group was consistent with those reported in previous studies, it failed to separate from placebo and therefore did not meet its primary end point of MADRS change at the end of the 6-week treatment period. (Zajecka et al., 2019). Most recently, Fava et al. (2020b) reported on the results of two further phase 3 studies of BUP/SAM in TRD: the FORWARD-4 and FORWARD-5 studies. In the FORWARD-5 study, BUP/SAM (2 mg/2 mg a day) was superior to placebo in terms of reduction in MADRS scores, while in the FORWARD-4 study the primary endpoint (change from baseline in MADRS at week 5 versus placebo) was not met (Fava et al., 2020b). Considering the findings of these trials, the Food and Drug Administration (FDA) did not support an initial application for the use of a fixed dose combination of BUP/SAM as an adjunctive treatment of MDD with FDA committees articulating concerns in their conclusions that 1) substantial evidence of effectiveness had not yet been demonstrated and 2) the data had not demonstrated a favourable benefit-risk profile to support approval (although samidorphan largely negates the MOR properties of buprenorphine, there remains some evidence of a mild opiate effect from the trials) (FDA, 2018). In work directly comparing the effect of samidorphan on the abuse potential of buprenorphine in the BUP/SAM combination in nondependent, recreational, adult opioid users BUP/SAM 2 mg/2 mg showed no abuse potential on the primary outcome, “at the moment” Drug Liking, as well as on key secondary measures such as Overall Drug Liking and Take Drug Again (Pathak et al., 2019). Although the incidence of euphoric mood (that may be suggestive of abuse potential) was 2- to 3- fold lower with BUP/SAM compared to buprenorphine alone, 18% in the BUP/SAM 2 mg/2 mg group still reported euphoric mood (Pathak et al., 2019). Further safety findings and robust assessment of BUP/SAM abuse potential are still needed.

While subtle MOR agonist effects of the BUP/SAM combination may be sufficient to modulate dysregulated opioidergic function, thereby improving symptoms in some individuals with depression, an alternative explanation is that antidepressant effects of the BUP/SAM combination may be driven by buprenorphine’s KOR antagonism (which is unaffected by samidorphan). To date, there are very limited published data on the antidepressant effects of specific KOR antagonists in MDD. In one study of the KOR selective antagonist CERC-501 (now known as JNJ-67953964) in TRD, mean differences of changes in HAMD-6 and MADRS were numerically but not statistically greater for CERC-501 than for placebo after 6-weeks, however, the study was terminated early due to slow enrolment and the small sample size (n = 8) prohibits any meaningful conclusions to be drawn (Fava et al., 2020a). A large phase 2a RCT designed to evaluate the efficacy of JNJ-67953964 compared to placebo when administered as adjunctive treatment in participants with MDD has since been completed (n = 181), however the results of this trial are yet to be published (ClinicalTrials.gov Identifier: NCT03559192). A randomised, double-blind, placebo-controlled, proof-of mechanism trial evaluating JNJ-67953964 as a treatment for anhedonia, demonstrated that in patients with anhedonia and a mood or anxiety disorder, a JNJ-67953964 treated group (10 mg/day over 8 week) (n = 45) exhibited significantly increased functional magnetic resonance imaging (fMRI) ventral striatal activation during reward anticipation in the Monetary Incentive Delay Task compared with placebo (n = 44) (Krystal et al., 2020). The finding that JNJ-67953964 influences brain function implicated in hedonic responses and that clinical anhedonia, as measured by the Snaith-Hamilton Pleasure Scale (SHAPS), was reduced following JNJ-67953964 relative to placebo treated subjects, suggests that JNJ-67953964 has the potential as an anhedonia therapy that may have therapeutic implications beyond the treatment of MDD. Finally, although there has been a focus on the development of KOR antagonists, one biotechnology company is currently developing salvinorin A, a KOR agonist (with other effects on dopaminergic, glutamatergic and cannabinoid systems as highlighted earlier (Taylor and Manzella, 2016)), with plans to examine its safety and efficacy in TRD (Revixia, 2021).

Compared to the limited number of clinical studies investigating the antidepressant effects of KOR specific antagonists, there is even less work examining the antidepressant potential of selective DOR agonists in humans. One major issue that has limited the development of DOR agonists as antidepressant candidates, is the pro-convulsive activity associated with some of these compounds, as highlighted in preclinical work (Broom et al., 2002a, Jutkiewicz et al., 2006a). In one small randomised, placebo-controlled pilot trial of the selective delta opioid receptor agonist AZD2327 in patients with anxious MDD (n = 22), after 4 weeks of treatment with AZD2327 3 mg BD, seven (54%) patients responded to AZD2327 and three (33%) responded to placebo (Richards et al., 2016). However, no significant main drug effect was found for either the HAM-D (p = 0.39) or the HAM-A (p = 0.15). Crucially, no epileptiform activity or seizures were observed in subjects, despite concerns from preclinical models. While the authors suggest increasing the dose of AZD2327 may have resulted in greater antidepressant/anxiolytic effects, this could increase the risk of seizures or other significant adverse events and any future study at higher doses would require close participant monitoring. Otherwise, building on preclinical work, DOR agonists that do not produce convulsions (Naidu et al., 2007, Vergura et al., 2008, Le Bourdonnec et al., 2008, Saitoh et al., 2011) may be preferable candidates that warrant further exploration as novel antidepressants.

Finally, NOP receptor antagonists have also attracted interest as potential therapeutic targets for the treatment of depression. In an 8-week, double-blind, placebo-controlled, proof-of-concept study evaluating LY2940094 (a selective and potent NOP receptor antagonist) as a novel oral medication for the treatment of MDD, following once daily oral dosing of LY2940094 at 40 mg (n = 68) the least-squares mean change from baseline in HAM-D total score was − 11.4 compared to − 9.8 for patients in the placebo treatment group (n = 61) (Post et al., 2016). The probability that LY2940094 was better than placebo was 82.9%, which was close to, but did not meet, the pre-defined proof-of-concept criterion (88% probability). To date, there are no other published clinical trial results examining the efficacy of NOP receptor antagonists in MDD.

## 6 Other antidepressant treatments and the opioid system

### 6.1 Tianeptine

Tianeptine is an atypical tricyclic-like antidepressant that has established efficacy and tolerability in MDD (Kasper and Olie, 2002). It shows relatively fast antidepressant and anxiolytic effects, including in individuals who are resistant to SSRI treatment (Woo et al., 2013), and has been reported to have a favourable side-effect profile compared with SSRIs and tricyclic antidepressants (McEwen et al., 2010). The primary molecular mechanism of tianeptine remained elusive for many years after its introduction as an antidepressant. Unlike other tricyclic antidepressants, tianeptine does not inhibit monoamine transporters, and although it has been shown to modulate N-methyl-D-aspartate (NMDA) and α-amino-3-hydroxy-5-methyl-4-isoxazolepropionic acid (AMPA) receptor function (McEwen et al., 2010), tianeptine does not appear to have measurable affinity for these glutamatergic nor monoamine neurotransmitter receptors. (McEwen et al., 2010, Svenningsson et al., 2007). More recently, tianeptine was identified as an efficacious MOR full agonist, binding to the human MOR with a Ki of 383 ± 183 nM (Gassaway et al., 2014). Tianeptine was also found to act as a full agonist at the DOR, although with much lower potency and was inactive at the KOR (Gassaway et al., 2014). This group further demonstrated that the MOR was required for the acute and chronic antidepressant-like effects of tianeptine in mice (Samuels et al., 2017). Although tianeptine also produced behavioural effects including analgesia and reward, its effects were distinct from morphine in that it did not produce tolerance or withdrawal. The authors question whether tianeptine may act as a biased agonist of the MOR and preferentially engage different signalling pathways than classical opioids and suggest that MOR downstream signalling cascades could be potential targets for novel antidepressant development (Samuels et al., 2017).

Unfortunately, there has been increasing controversy surrounding the use of tianeptine with reports of patients abusing the drug at doses beyond the therapeutic range, for prolonged periods, leading to dependence (Springer and Cubala, 2018, Rushton et al., 2021). Prominent features associated with tianeptine abuse and dependence include acute euphoria and withdrawal effects like those seen with typical opioid drugs which suggests MOR activation is also likely responsible for its abuse potential (Springer and Cubala, 2018, Rushton et al., 2021).

### 6.2 Ketamine and Esketamine

Over the last two decades, the dissociative anaesthetic agent ketamine, an uncompetitive NMDA receptor antagonist, has emerged as a novel antidepressant (Jelen and Stone, 2021). Multiple short term RCTs have established rapid (within 24 h) and significant efficacy of intravenous racemic (R,S)-ketamine administered at subanaesthetic doses (0.5–1.0 mg/kg)), and of intranasal (S)-ketamine, esketamine (Spravato®), (28 – 84 mg) in adults with TRD (Kryst et al., 2020, McIntyre et al., 2020, Papakostas et al., 2020). It has been proposed that the antidepressant effects of ketamine are mediated through blockade of NMDA receptors located on γ-aminobutyric acid (GABA)-ergic interneurons that normally act to suppress glutamate release from pyramidal neurons (Krystal et al., 2019). This disinhibition of pyramidal neurons appears to cause an acute glutamate surge (Moghaddam et al., 1997, Abdallah et al., 2018), subsequent activation of post-synaptic AMPA receptors, resulting in activation of neuroplastic signalling pathways and synaptogenesis (Lener et al., 2017). However, in addition to glutamatergic effects, ketamine also acts on a range of other neurotransmitter systems including monoaminergic, cholinergic and opioid systems which may also contribute to its antidepressant effects (Jelen and Stone, 2021).

Focussing on ketamine’s effects on the opioid system, ketamine has micromolar affinity for the MOR, KOR and even less so for the DOR (Ki = 42.1, 28.1, and 272 mΜ, respectively) (Hirota et al., 1999, Zanos et al., 2018). Clinical and preclinical work have demonstrated that ketamine produces opioid receptor-dependent analgesia (Ryder et al., 1978, Finck and Ngai, 1982, Pacheco Dda et al., 2014), potentiates opioid analgesic effects (Baker et al., 2002), reduces opioid tolerance and opioid-induced hyperalgesia (Koppert et al., 2003) and at higher doses produces MOR-dependent respiratory depression (Sarton et al., 2001). However, less is known about the role of the opioid system for the lower doses of ketamine used for antidepressant effects. Recently, it was demonstrated that opioid system activation may be necessary for the acute therapeutic effects of ketamine. In a small, double-blind randomised crossover trial in TRD (n = 12 completers), pre-treatment with naltrexone (a MOR/KOR antagonist) significantly attenuated the antidepressant and anti-suicidal effects of ketamine (Williams et al., 2018, Williams et al., 2019). While other pilot work (n = 5) has found that naltrexone pre-treatment did not impact the antidepressant effects of ketamine in depressed individuals with comorbid alcohol use disorder (Yoon et al., 2019), both this study and those by Williams et al. were limited by small sample sizes and only investigated acute effects of opioid receptor antagonism. Another study, using data from 2 phase III studies of esketamine, examined whether the single nucleotide polymorphism rs1799971 (A118G) of *OPRM1*, which is known to alter MOR agonist-mediated responses affected the antidepressant and dissociative responses to esketamine (Saad et al., 2020). No significant genotype effect on reductions in MADRS total score or on dissociative responses in patients with TRD treated with esketamine and oral antidepressant which did not suggest the antidepressant effect of esketamine was mediated by MOR activation. Larger clinical replication studies are needed to clarify the role of the opioid system in both the acute and post-acute phase of ketamine and esketamine’s antidepressant effects.

Findings from preclinical studies investigating the role of the opioid system in ketamine’s antidepressant-like effects have also not been consistent. One study found that naltrexone pre-treatment did not block the effects of ketamine in chronic social defeat stress and inflammation induced rodent models of depression (Zhang and Hashimoto, 2019). Nonetheless, a subsequent study in rodents demonstrated that activation of opioid receptors is ‘necessary but not sufficient’ for ketamine to reduce depressive-like behavioural symptoms and to reduce activity in the lateral habenula, a critical anti-reward centre implicated in the pathophysiology of depression (Klein et al., 2020). In this study, although ketamine did not appear to act as a mu-opioid agonist per se, some MOR activity was necessary for NMDA receptor antagonism (Klein et al., 2020). As MOR and NMDA receptors display colocalization across certain brain regions (Rodriguez-Munoz et al., 2012) and opioid receptor activity can modulate NMDA receptor activation (Kow et al., 2002), this supports the notion of ‘crosstalk’ between the MOR and glutamatergic receptor systems (Chartoff and Connery, 2014), that may have a role in mediating ketamine’s antidepressant effects. Recently, a study in mice also found that naltrexone pre-treatment attenuated the acute and sustained effects of ketamine- induced reductions in immobility in the TST (Zhang et al., 2021). Additionally, it has been reported that intra-medial prefrontal cortex injections of naltrexone and β-endorphin neutralizing antibody both block ketamine-induced reductions in immobility in the FST and antidepressant-like effects in the female urine sniffing test in male rats (Jiang et al., 2021). Together, these data further suggest the MOR system may contribute to the antidepressant effects of ketamine, but these effects may be permissive rather than direct effects. Other work, focussing on the KOR system, has demonstrated that acute treatment with ketamine causes internalisation of KORs in HEK293 cells and pre-treatment with the selective KOR antagonist LY2444296 blocks the antidepressant-like effects of ketamine on the forced-swim test (Jacobson et al., 2020b). Furthermore, ketamine administration blocked KOR agonist induced increases in activity in the lateral habenula (Jacobson et al., 2020b). Although speculative, this suggests that ketamine administration may lead to reductions in KOR signalling, resulting in reductions in negative and pro-depressive feelings associated with KOR agonism, thereby contributing to the clinical antidepressant effects of ketamine (Alexander et al., 2021).

Finally, ketamine has known abuse liability, and some have raised concerns for its prolonged and widespread therapeutic use (Schatzberg, 2014). Crucially, ketamine is a racemic mixture, composed of (S)- and (R)- enantiomers that may individually exert antidepressant effects via distinct mechanisms with potential differences in abuse potential (Jelen et al., 2021). In a key rodent study, while (S)-ketamine and (R)-ketamine both bound to and activated MORs and KORs, (S)-ketamine showed higher affinity and potency than (R)-ketamine (Bonaventura et al., 2021). Furthermore, antidepressant-like doses of (S)-ketamine, but not (R)-ketamine, induced locomotor activity, psychomotor sensitization, induced conditioned place preference in mice, and selectively increased metabolic activity and dopamine tone in medial prefrontal cortex (mPFC) of rats, in an opioid receptor dependent manner (Bonaventura et al., 2021). Together this suggests that racemic ketamine’s abuse liability in humans may be primarily driven by the pharmacological effects of its (S)-enantiomer. This has important implications for the clinical use of (S)-ketamine, especially in individuals with comorbid substance use disorders. If clinical trials of (R)-ketamine (PCN-101) (EudraCT Number: 2020–005457–25) demonstrate efficacy in TRD, (R)-ketamine may prove to have a favourable benefit-risk treatment profile over the (S)- enantiomer, especially for those patients with comorbid substance use disorders.

### 6.3 Esmethadone (REL-1017)

Esmethadone is the d-isomer of the opioid drug methadone. Esmethadone has been described as an opioid inactive isomer of racemic methadone that acts as a low-affinity, low-potency NMDA receptor antagonist (Fava et al., 2022). Although it has 20 times lower affinity for MOR than levomethadone, this is still significantly (70 times) higher than its affinity for NMDA receptors (Gorman et al., 1997, Codd et al., 1995). In healthy subjects, esmethadone has been found to increase circulating levels of brain-derived neurotrophic factor (BDNF) (De Martin et al., 2021), a neurotrophin widely expressed in the central nervous system that exhibits important effects on neural plasticity, and in a recent phase II placebo-controlled, randomised clinical trial of patients with TRD, 1-week of treatment with esmethadone at doses of 25 or 50 mg exhibited clear efficacy at day 4, 7 and 14 (7 days after the last dose) (Fava et al., 2022). Although there were no characteristic opiate effects or withdrawal symptoms reported after day 7 in this study this does not necessarily mean esmethadone is devoid of opiate activity. In a press release of a study comparing the “likeability” of esmethadone (25, 75 and 150 mg) with oxycodone 40 mg and placebo in recreational opioid users, placebo scored at 51.7 (50 is neutral and higher numbers are more likeable) with esmethadone 25, 75, and 150 mg rated at 53.0, 58.2, and 64.9, respectively, compared with oxycodone 40 mg, that had a rating of 85 (Therapeutics, 2021). However, it has been argued the oxycodone dose of 40 mg might be considered inordinately high as the lower dose of 20 mg is known to be “likeable” by experienced recreational drug users and might have provided valuable information about comparative drug abuse liability of esmethadone (Nemeroff, 2022). Results from a larger phase III study of esmethadone in TRD are awaited and further mechanistic work is needed to clarify whether the potential antidepressant effects of esmethadone are mediated via the opioid system.

### 6.4 Neurokinin-1 receptor antagonists

The neurokinin-1 (NK1) receptor and its endogenous neuropeptide Substance P are expressed in high density in brain regions implicated with the pathophysiology of stress and depression including the cingulate cortex, caudate, putamen, nucleus accumbens, hippocampus, amygdala, various hypothalamic areas, as well as the dorsal raphe nucleus, and locus coeruleus (Ebner and Singewald, 2006). Substance P and NK1 receptors interact with serotonergic and noradrenergic pathways (Conley et al., 2002, Maubach et al., 2002) but are also associated with the opioid system. NK1 receptors and MOR receptors often colocalise (Aicher et al., 2000) and inhibition of substance P release has been hypothesised as one of the mechanisms for opioid drugs (Chen et al., 2014). Preclinical studies of NK1 antagonists supported their potential for the treatment of depression and anxiety (McLean, 2005) and antidepressant efficacy was subsequently demonstrated in clinical studies of three different NK1 antagonist compounds (Kramer et al., 1998, Kramer et al., 2004, Chappell, 2002). Unfortunately, after the failure of a large Phase III program using a lower dose nanoformulation (Keller et al., 2006), the development of NK1 antagonists for depression was abandoned. However, this failure may have been due to an inadequate understanding of the relationship between brain NK1 receptor occupancy and clinical response and that higher doses should have been systematically evaluated (Rupniak and Kramer, 2017). It remains to be seen if future work to determine novel mechanisms underlying potential antidepressant effects of NK1 antagonists, including any interactions with the opioid system, may help to restimulate interest in the development of these compounds for depression.

## 7 Opioid system and antidepressant effects: neurochemical and molecular mechanisms

### 7.1 Monoaminergic systems

There are several functional interactions between opioid receptors and the monoaminergic systems that are relevant to mood control (Lutz and Kieffer, 2013). In vivo microdialysis studies have shown that activation of MORs, expressed in the dorsal raphe nucleus, suppresses GABAergic interneuron inhibition, resulting in a disinhibition of serotoninergic neurons and increased serotonin (5-HT) release in brain areas including the frontal cortex and various limbic regions (Tao and Auerbach, 1995, Tao and Auerbach, 2002, Fadda et al., 2005). Opioid receptors also play a role in regulating mesolimbic dopaminergic activity with activation of MORs and DORs in the ventral tegmental area, enhancing dopamine release in the nucleus accumbens (Pentney and Gratton, 1991, Devine et al., 1993). In contrast, KOR activation reduces dopamine release in the nucleus accumbens (Spanagel et al., 1992, Carlezon et al., 2006) and induces a negative mood state (Bruijnzeel, 2009), whereas selective blockade of KORs markedly increases dopamine release (Spanagel et al., 1992) and prevent the development of anhedonic-like states (Carlezon and Krystal, 2016). Together, these pro-serotoninergic /pro-dopaminergic effects of MOR/DOR agonists and KOR antagonists could ultimately contribute to potential antidepressant effects of such opioidergic agents.

### 7.2 Hypothalamic-pituitary-adrenal (HPA) axis

The stress responsive HPA axis has been increasingly implicated in the pathophysiology of depression (Keller et al., 2017). The axis consists of direct influences and feedback inhibition loops involving the hypothalamus, the pituitary gland, and the adrenal glands via corticotrophin releasing factor (CRF), vasopressin, adrenocorticotrophic hormone (ACTH) and glucocorticoids (i.e. cortisol). Crucially, it appears abnormalities of HPA functioning in depression are related to reduced feedback inhibition by endogenous glucocorticoids, resulting in hyperactivity of the axis (Pariante, 2006). There is evidence to suggest the endogenous opioid system plays an important role in modulating behavioural and HPA axis responsivity to stress (Drolet et al., 2001, Bilkei-Gorzo et al., 2008), with β-endorphin and dynorphin exerting tonic inhibition and stimulation of HPA activity by acting on the MOR and KOR, respectively (Zhou and Leri, 2016).

In primates, self-administration of the potent MOR agonist fentanyl has been shown to inhibit HPA activity with a reduction in plasma cortisol and ACTH (Broadbear et al., 2004). Interestingly, this study found that ketamine administration had a similar inhibitory effect on ACTH and cortisol secretion. In contrast, a different group found that the MOR antagonist naltrexone (but not the DOR selective antagonist naltrindole) increased ACTH and cortisol levels (Williams et al., 2003). Other work in primates demonstrated that acute administration of a KOR agonist led to a dose dependent increase in plasma ACTH and cortisol levels (Pascoe et al., 2008). Importantly, these stimulatory effects were blocked by a selective KOR antagonist.

In humans, administration of the MOR agonist morphine and the MOR agonist/ KOR antagonist buprenorphine also leads to suppression of ACTH and glucocorticoid secretion, alongside diminished cortisol response to psychosocial stress (Allolio et al., 1987, Bershad et al., 2015). In contrast, administration of a KOR agonist caused a dose dependent increase in cortisol release in man (Ur et al., 1997). Considering these findings, the stress-dampening effect of MOR agonists / KOR antagonists and inhibition of the HPA axis may be another mechanism that could contribute to the antidepressant effects of these agents.

### 7.3 Brain-derived neurotrophic factor (BDNF)

BDNF and its tyrosine kinase receptor (TrkB) play a critical role in processes that include neuronal maturation, synapse formation and synaptic plasticity (Park and Poo, 2013). Furthermore, BDNF-TrkB signalling has been implicated in the pathophysiology of depression and may be responsible for the therapeutic effects of current antidepressant treatments (Duman and Monteggia, 2006, Dwivedi, 2009). Specifically, preclinical findings suggest increased BDNF-TrkB signalling in hippocampal and prefrontal cortex regions may drive the antidepressant response to conventional antidepressants (Adachi et al., 2008, Schmidt and Duman, 2010, Yang et al., 2020). Importantly, opioid receptors also regulate BDNF activity and regional increases in BDNF expression may be another mechanism of action for the antidepressant-like effects of certain opioid modulating agents. In rodents, central administration of endogenous opioid peptides significantly upregulates BDNF expression in the frontal cortex and hippocampus through activation of MORs and DORs (Zhang et al., 2006). Further rodent work has shown that administration of DOR agonists, at doses that produced antidepressant-like effects, resulted in acute upregulation of BDNF expression in brain regions including the frontal cortex, hippocampus, and amygdala (Torregrossa et al., 2004, Torregrossa et al., 2006). Finally, the selective KOR antagonist nor-BNI also produced antidepressant-like behavioural effects in rats, associated with upregulated BDNF expression in the hippocampus and amygdala (Zhang et al., 2007). However, research examining the relationship between BDNF and antidepressant-like effects of opioidergic agents has been limited to animal models, and the relevance in humans remains to be determined.

### 7.4 Intracellular signalling

There are several key intracellular signalling pathways implicated in MDD that may be directly or indirectly modulated by opioid receptors including extracellular signal-related kinase/mitogen-activated protein kinase (ERK/MAPK), cyclic adenosine monophosphate (cAMP)-response element binding protein (CREB) and activator protein-1 (AP-1) (Puryear et al., 2020).

The ERK/MAPK signalling pathway plays a key role in regulating protein synthesis required for synaptic development and plasticity (Wang and Mao, 2019). Furthermore, phosphorylated ERK1/2 (pERK1/2) is upregulated in response to both conventional and novel rapid acting antidepressants (First et al., 2013, Gourley et al., 2008, Lepack et al., 2016, Qi et al., 2008). Acute MOR activation similarly leads to rapid activation of ERK signalling in vitro (Zheng et al., 2008, Li and Chang, 1996, Belcheva et al., 2005) and acute MOR or DOR activation increases both mitogen-activated protein kinase kinases (MEK1/2) and ERK1/2 activity in the rat cortex (Asensio et al., 2006). KOR-mediated ERK1/2 phosphorylation has also been demonstrated in vitro (Belcheva et al., 2005) and in vivo in mice (Bruchas et al., 2008). Although MOR, DOR and KOR activation all modulate ERK/MAPK activity, and have a role in neuroplastic mechanisms (Al-Hasani and Bruchas, 2011), the behavioural consequences are not entirely clear. Stress-induced activation of the dynorphin-KOR system appears to activate ERK/MAPK signalling (Bruchas et al., 2008) and administration of KOR agonists also activates ERK while inducing anhedonia-like behaviours (reversed by KOR antagonists) (Potter et al., 2011). Overall, these data demonstrate that opioidergic agents administered acutely modulate ERK1/2 in a similar manner to treatment with conventional and rapid acting antidepressants, however, it should not be assumed that increased ERK/MAPK signalling is directly associated with antidepressant effects. While modulating ERK/MAPK dependent plasticity may be an important opioidergic antidepressant mechanism, these effects are complex, receptor and region specific, and likely context dependent (Puryear et al., 2020).

The transcription factor CREB, known for its involvement in learning and memory, is an additional intracellular target of opioid receptor modulation that may be dysregulated in MDD (Carlezon et al., 2005). CREB activity is modulated by stress and can have differential roles- sometimes beneficial, sometimes detrimental- depending on the brain region involved (Carlezon et al., 2005). Hippocampal expression of CREB is reduced in response to stress exposure (Alfonso et al., 2006) and chronic administration of a range of antidepressant treatments upregulate CREB expression in this brain region (Nibuya et al., 1996), including the MOR agonist tianeptine (Lee et al., 2017). In contrast, there is evidence that stressors activate CREB in the nucleus accumbens (Pliakas et al., 2001, Bruchas et al., 2007) and CREB-mediated transcription in this region may regulate dysphoric states (Knoll and Carlezon, 2010). Disruption of CREB function in the nucleus accumbens produces antidepressant-like effects in rodents (Pliakas et al., 2001) alongside reductions in dynorphin expression (Carlezon et al., 1998). Elevated CREB activity within the nucleus accumbens increases immobility in the forced swim test and KOR antagonists attenuate this effect, suggesting CREB-mediated induction of dynorphin contributes to these depressive-like effects (Pliakas et al., 2001). Additional work has demonstrated other stressors including immobilisation, foot-shock and social defeat also alter CREB and KOR function in the nucleus accumbens (Shirayama et al., 2004, Muschamp et al., 2011, Donahue et al., 2015), supporting the hypothesis that KOR antagonists attenuate depressive-like effects of elevated CREB within the nucleus accumbens, providing further mechanistic rational for the investigation of KOR antagonists for the treatment of depressive illness in humans.

AP-1 is a transcription factor, composed of immediate early gene proteins belonging to the Jun and Fos families, and is major downstream target of BDNF and ERK/MAPK signalling cascades, that regulates gene expression associated with neuroplasticity (Chottekalapanda et al., 2020). It has recently been demonstrated that the specific induction of AP-1 by chronic SSRI treatment is essential in initiating a transcriptional program that modulates the expression of key neuronal plasticity genes which then leads to an antidepressant response in vivo (in rodent models) (Chottekalapanda et al., 2020). MOR agonists have also been shown to acutely induce AP-1 activity (Shoda et al., 2001, Bilecki et al., 2004), which is attenuated by MAPK inhibitors (Shoda et al., 2001). Although speculative, activation of AP-1 by opioidergic agents may be another mechanism to accelerate or potentiate antidepressant responses by triggering neuroplasticity.

## 8 Conclusion

In this review, integrating findings across preclinical and clinical studies, we have highlighted key roles for the opioid system in depression. Initial evidence from animal data has demonstrated distinct functions of the opioid receptor subtypes, MOR, DOR, KOR, and NOP receptor, in regulating mood-related processes including reward, motivation and social behaviours, with pharmacological studies suggesting antidepressant potential of MOR/DOR agonist and KOR/NOP receptor antagonist compounds in rodent models of depression. Evidence from direct experimental work in humans has implicated the opioid system in emotional processing and demonstrated dysregulation of this system in depression. Specifically, dysregulated MOR function in regions implicated in the regulation of mood, cognitive control, and emotional processing (amygdala, insula, hippocampus, ventral striatum, and cingulate cortices), may contribute to the alterations in mood, dysfunctional processing of negative social interactions and social anhedonia, characteristically seen in depression. In parallel, increasing evidence from recent experimental medicine studies indicate that mu-opioid modulation may be involved in antidepressant responses, via placebo effects based on reward and reinforcement principles. Clinical studies of low doses of MOR agonists and MOR/KOR agonist-antagonist combinations (BUP/SAM) in MDD have provided early indication of antidepressant efficacy, while minimising the risk of abuse liability. Advances in the understanding of the functions of individual opioid receptor subtypes and the development of selective opioid ligands, including DOR selective agonists, and KOR and NOP receptor selective antagonists, has brought promise for the potential of opioidergic agents as viable treatment options for MDD, without the risk of addiction. Compared to MOR, the DOR, KOR and NOP receptor systems remain relatively understudied in humans, and further clinical studies are needed to explore questions of safety, efficacy, and therapeutic mechanisms of compounds targeting these receptors. As ketamine becomes more widely prescribed for depression, there is ongoing controversy surrounding the specific role of the opioid system in modulating ketamine’s antidepressant effects. Experimental medicine studies that directly examine potential opioid mechanisms in the antidepressant action of ketamine (NCT04977674), conventional antidepressants, placebos, and placebo effects, may elucidate novel functions of the opioid system that ultimately allow us to maximise therapeutic efficacy of existing treatment strategies and inform the development of future opioid-modulating antidepressants that could provide an improved option, with distinct clinical benefits, for those patients suffering with difficult-to-treat depression.

## Funding

This report represents independent research funded by the National Institute for Health Research (NIHR) Biomedical Research Centre at South London and Maudsley NHS Foundation Trust and King’s College London. The views expressed are those of the authors and not necessarily those of the NHS, the NIHR, or the Department of Health.

**Luke Jelen:** Employed by King’s College London. Dr Luke A Jelen is supported by a Medical Research Council (MRC) Clinical Research Training Fellowship (MR/T028084/1).

**James Stone:** Employed by The University of Sussex.

**Allan Young:** Employed by King’s College London; Honorary Consultant SLaM (NHS UK). Grant funding (past and present): 10.13039/100000025NIMH (USA); 10.13039/501100000024CIHR (Canada); 10.13039/100009670NARSAD (USA); 10.13039/100007123Stanley Medical Research Institute (USA); 10.13039/501100000265MRC (UK); 10.13039/100004440Wellcome Trust (UK); 10.13039/501100000395Royal College of Physicians (Edin); 10.13039/501100000374BMA (UK); UBC-VGH Foundation (Canada); WEDC (Canada); CCS Depression Research Fund (Canada); 10.13039/501100000245MSFHR (Canada); 10.13039/100006662NIHR (UK). Janssen (UK). No shareholdings in pharmaceutical companies.

**Mitul Mehta:** Employed by King’s College London. Grant funding (past 3 years): Takeda, Johnson & Johnson, Lundbeck, WellcomeTrust (212952/Z/18/Z; 200102/Z/15/Z), 10.13039/501100000265MRC (MR/R005931/1; MR/R005885/1; MR/S003444/1), 10.13039/100006662NIHR (CRF-2016-10023).

## Declaration of Competing Interest

**Dr Luke Jelen:** The author declared no potential conflicts of interest with respect to the research, authorship, and/or publication of this article. **Dr James Stone:** Dr Stone reported grants from Protexin Probiotics International, personal fees from Janssen, and grants from Takeda. **Professor Allan Young:** Paid lectures and advisory boards for the following companies with drugs used in affective and related disorders: Astrazenaca, Eli Lilly, Lundbeck, Sunovion, Servier, Livanova, Janssen, Allegan, Bionomics, Sumitomo Dainippon Pharma, COMPASS, Sage, Novartis. Consultant to Johnson & Johnson. Consultant to Livanova. Received honoraria for attending advisory boards and presenting talks at meetings organised by LivaNova. Principal Investigator in the Restore-Life VNS registry study funded by LivaNova. Principal Investigator on ESKETINTRD3004: “An Open-label, Long-term, Safety and Efficacy Study of Intranasal Esketamine in Treatment-resistant Depression.” Principal Investigator on “The Effects of Psilocybin on Cognitive Function in Healthy Participants.” Principal Investigator on “The Safety and Efficacy of Psilocybin in Participants with Treatment-Resistant Depression (P-TRD).” UK Chief Investigator for Novartis MDD study MIJ821A12201. Grant funding (past and present): NIMH (USA); CIHR (Canada); NARSAD (USA); Stanley Medical Research Institute (USA); MRC (UK); Wellcome Trust (UK); Royal College of Physicians (Edin); BMA (UK); UBC-VGH Foundation (Canada); WEDC (Canada); CCS Depression Research Fund (Canada); MSFHR (Canada); NIHR (UK). Janssen (UK). No shareholdings in pharmaceutical companies. **Professor Mitul Mehta:** Has acted as a Consultant for Lundbeck and Neurocrine.
